# How workplace incivility leads to work alienation: A moderated mediation model

**DOI:** 10.3389/fpsyg.2022.921161

**Published:** 2022-09-02

**Authors:** Bingnan Xia, Xiaochen Wang, Qing Li, Yuzhen He, Wei Wang

**Affiliations:** ^1^School of Business Administration, Zhejiang Gongshang University, Hangzhou, China; ^2^School of Marxism, Communication University of Zhejiang, Hangzhou, China; ^3^Hangzhou Zhongxing Hospital, Hangzhou, China

**Keywords:** workplace incivility, interpersonal trust, work alienation, career resilience, social exchange

## Abstract

Workplace incivility remains a prevailing issue and has significant potential for harmful consequences. This study aims to investigate the influencing mechanism of workplace incivility on work alienation from the perspective of targets. Based on the social exchange theory, our research examines the role of interpersonal trust as a mediator along with the moderator of career resilience in the said association. Through a two-wave-time-lagged quantitative research design, a sample of 315 nurses from China was investigated with questionnaires on workplace incivility, work alienation, interpersonal trust, and career resilience. The results indicated that workplace incivility was positively related to work alienation with interpersonal trust as a mediator. Workplace incivility caused a decline in interpersonal trust, which led to work alienation. Career resilience buffered such an impact. High career resilience weakened the association linking workplace incivility to interpersonal trust. Organizations should pay more attention to workplace incivility and consider empowering nurses’ career resilience, which could alleviate the negative impact of workplace incivility.

## Introduction

Workplace incivility, such as “talking down to others and making demeaning remarks” (Pearson and Porath, 2009, p. 57), is both pervasive and damaging, consistent with research demonstrating its negative impact on targets’ physical and mental health, as well as work efficiency (Nicholson and Griffin, 2015). Healthcare settings are not immune (Samad et al., 2020). Spence Laschinger et al. (2009) reported that 67.5% of the nurses were exposed to workplace incivility from supervisor nurses and 77.6% from coworkers. The COVID-19 outbreak brought sudden and profound changes to many health care settings. Nurses, as a group of indispensable first respondents safeguarding public health, are not only working on constant moves dealing with medical emergencies (Grimm, 2020), but also are confronting the danger of being infected (Osterdahl et al., 2020). This would inevitably increase their workload and workplace stressors, which in turn leads to an increase in uncivil behaviors (Roberts et al., 2011; Oyeleye et al., 2013). The internationally recognized phenomenon of incivility remains a prevailing conundrum in the field of nursing (Fida et al., 2018; Blackstock et al., 2022). Thus, suggesting the urgency of understanding incivility in healthcare and how incivility leads to critical healthcare outcomes (Andel et al., 2022).

Studies have shown that workplace incivility may trigger job burnout (Kang et al., 2017), resulting in reduced productivity (Hutton and Gates, 2008), and even turnover intention (Kanitha and Naik, 2021). Specifically, before taking action to leave their current jobs, individuals may start with resenting their jobs, by which time a sensation of getting away from work, such as work alienation, may grow (Dutton et al., 2010). Furthermore, as studies have revealed that the main perpetrators are nurses in a senior position to those being bullied and colleagues who are established staff members (Wilson, 2016), we focus on instigators of incivility primarily from within the organization (from supervisors or coworkers). Based on previous studies and taking a perspective of targets of incivility, we explore the influencing mechanism and boundary conditions of workplace incivility on nurses’ mental states at work.

In order to obtain a more fine-grained understanding of the mechanisms through which workplace incivility triggers nurses’ feelings of alienation, this study introduced interpersonal trust. Trust serves as the foundation for social exchange relationships and is key factor in the emergence and deepening of such relationships (Konovsky and Pugh, 1994; Colquitt et al., 2012). Interpersonal trust has been examined as a vital component explaining diverse dynamics and interactions in organizations (Yang et al., 2009; Kim and Park, 2019). It involves reciprocated interpersonal care and concern for another person (Rempel et al., 1985) and is associated with favorable outcomes for employees and the organization (Altuntas and Baykal, 2010), for instance, organizational commitment (Baek and Jung, 2015) and job satisfaction (Guinot et al., 2014). Moreover, research suggests that trust may be broken by actual misbehaviors and transgressions (Kim et al., 2013; Lanaj et al., 2018), leading to feelings of insecurity, uncertainty, and anxiety (Carlson and Perrewé, 1999). Social Exchange Theory (SET) proposes that interpersonal relations function according to a potential reciprocity norm (Gouldner, 1960), which could affect mutual trust. The high-quality interpersonal relationship not only lessens one’s work pressure but also broadens a sense of accomplishment and meaningfulness (Ali et al., 2021). However, workplace incivility violates norms of respect (Schilpzand et al., 2016), thereby undermining harmonious interpersonal relations in the workplace. Such strained interpersonal interactions tend to increase anxiety and tension (Guinot et al., 2014), leaving targets feeling empty and helpless and gradually losing the meaning of work (Wang et al., 2019). Further, it can arouse and reinforce their sense of estrangement from the work and the work context (Usman et al., 2020). Hence, we consider interpersonal trust a powerful mechanism of work alienation provoked by uncivil behaviors in the workplace. And its role in this relationship has not been studied. From the perspective of social exchange theory, this study seeks to reveal the mediating role of interpersonal trust between workplace incivility and nurse work alienation.

Furthermore, since civility interaction is a latent need, it focuses on the degree of individual perception (Yan and Cao, 2019). Due to the differences in individuals’ perception and interpretation of incivility, their corresponding psychological mechanisms and behavioral reactions are not the same. Uncivil behaviors are hostile exchanges that develop among organization members (Andersson and Pearson, 1999). Studies have indicated that the exchange process in SET may vary by individual differences (Cropanzano and Mitchell, 2005; Shore et al., 2009). To examine the differential exchange processes of incivility, we suggest that career resilience, the ability to “bounce back” from less optimal or encouraging career circumstances (London, 1983), is the boundary condition for the adverse effects of workplace incivility. Career resilience is considered to be the key to overcoming career stress (London and Mone, 1987). Not only does it provide individuals with the psychological freedom and flexibility to quickly recover from negative social interactions and the confidence in overcoming occupational adversity (Lent and Brown, 2013), but it also motivates them to achieve active learning and growth by conquering challenges (Youssef and Luthans, 2007; Lyons et al., 2015). In addition, some indirect evidence supports that resilience has beneficial effects on social exchange (Meng et al., 2019) and may contribute to improving interpersonal adaptation (Block and Kremen, 1996) and relationships (Waite and Richardson, 2004). In contrast to the group of nurses with weak career resilience, the group of nurses with high level of resilience, when encountering uncivil workplace behavior, are capable of adjusting their mindset quickly and reducing the adverse consequences (Jiang et al., 2021), especially the ones resulting from their optimistic assumptions for others, and mentally shield themselves from being alienated. This implies that career resilience can contribute to reducing the sense of work alienation that incivility targets experience.

The contribution of this study lies upon the novel perspective of incivility targets under the theoretical framework of social exchange theory. In particular, we establish the mediating role of interpersonal trust to investigate workplace incivility and work alienation in the medical system. Also, we verify the moderating role of career resilience in the relationship between workplace incivility and interpersonal trust. Furthermore, we test the moderating effect of career resilience on interpersonal trust’s mediating effect, forming a moderated mediation model to reveal how and when workplace incivility affects nurses’ work alienation. Results from the above generated have significantly extended the research of mistreatment in the workplace, thus highlighting the importance of developing workplace harmony with the utmost labor efficiency.

## Theory and hypotheses

### Workplace incivility and work alienation

Workplace incivility refers to a low-intensity deviant behavior that violates norms for mutual respect with ambiguous intent to harm the target, as a reflection of the social exchange relationships that develop among organization members (Andersson and Pearson, 1999). SET suggests that interpersonal interactions are guided by an underlying norm of reciprocity (Gouldner, 1960). Uncivilized behavior is interactive since uncivil behaviors from the instigator(s) would cause attitudinal or behavioral changes in the target(s) (Cortina et al., 2013). Those targets may experience boredom, loneliness, and frustration at work (Gallus et al., 2014), which could trace back to the reduction of working motivation and enthusiasm and, in the worst case, resulting in a regression within the workforce (Woo and Kim, 2020).

Accordingly, individuals may feel separated from work, resulting in work alienation—a state of psychological separation from work insofar as work is perceived to lack the potential for satisfying one’s salient needs and expectations (Banai et al., 2004). Work alienation reflects targets’ view of the connection between work and themselves and can produce a sense of incomprehensibility about their job and its importance (Nair and Vohra, 2009). It leads to emptiness and meaninglessness (Rokach, 2004; Peng et al., 2022), triggers emotional exhaustion (Khan et al., 2019) and burnout (Usman et al., 2020), and reduces wellbeing (Sarwar et al., 2022). In addition, the main cause of work alienation is that job cannot meet the needs and expectations of the individual (Yu et al., 2021). Existing research has demonstrated that spiritual and laissez-faire leadership in organizations are antecedents of job alienation (Usman et al., 2020; Ali et al., 2022). Research in this vein insinuates that interactive organizational factors (e.g., workplace incivility) are better predictors of alienation (DiPietro and Pizam, 2008).

Complying with the rules of minimum respect is one of the main rules of a social society (Kavakli and Yildirim, 2022). As a typical type of deviant behavior in the workplace, workplace incivility violates the norms of reciprocity (Wu et al., 2014) and conveys interpersonal cues such as rudeness, discourtesy, and a lack of concern for others (Schilpzand and Huang, 2018). When individuals perceive that they have been mistreated against expectations, they are prone to be overwhelmed in interpersonal interactions and unable to establish positive social relationships (Wang et al., 2019). After constantly undergoing this inability to interact effectively, targets may neglect the meaning of work and self-worth, thus finding themselves not identifiable with their work or organization psychologically, resulting in a sense of alienation from work (Shantza et al., 2014). Therefore, we propose the following hypothesis.

**Hypothesis 1.** Workplace incivility is positively related to work alienation.

### Mediating role of interpersonal trust

Social Exchange Theory argues that trust is essential to the development and deepening of social exchange relationships (Blau, 1964; Colquitt et al., 2014). Interpersonal trust is the extent to which a person is confident in, and willing to act on the basis of the words, actions, and decisions of another (McAllister, 1995). It reflects positive expectations about and a willingness to act upon the words and intentions of an exchange partner. Individuals are generally expected to work together to achieve common goals in organizational settings (Scott et al., 2013). Uncivil behaviors violate this expectation and erode the interpersonal benevolence or goodwill required to build trust (Gill and Sypher, 2009). According to SET, social exchanges, with reciprocity as a premise, affect people’s trust and unity, and positive reciprocity interaction can promote interpersonal trust (Mitchell and Ambrose, 2007). Workplace incivility, as a form of interpersonal mistreatment (Lim and Cortina, 2005), expresses negative information such as contempt and disrespect to targets and destroy the premise of social exchange between individuals. It would be hard to develop a sense of reciprocal care and concern in an environment where communications were rude or disrespectful (Colquitt et al., 2012). When targets feel offended, they may lose resources such as positive relationships; hence they experience less interaction in the workplace.

Negative evaluations of work relations are a significant cause of work alienation (Poole and Regoli, 2006). Incivility may lower targets’ trust in others and expectations as they experience reciprocity imbalance. People are more likely to participate in social exchanges with persons they trust since sharing resources with others might make them fragile (Peng et al., 2014). When interpersonal trust has been breached, individuals are typically hesitant to form attachments and interact with others (Kramer, 1999). For these reasons, victims of incivility may become indifferent to their surroundings and lack passion for their work, which leads to a weaker sense of responsibility, obligation and lower levels of job involvement (Welbourne and Sariol, 2017). In this vein, intense nerves and heavy pressure induced by uncivil behaviors may harm the harmonious relationship and destroy trust between individuals, resulting in work alienation. Therefore, we propose:

**Hypothesis 2.** Interpersonal trust acts as a mediator between workplace incivility and work alienation.

### Moderating role of career resilience

Despite the wide application of social exchange theory (Gouldner, 1960), researches demonstrate that there are individual differences in the process of social exchange (Shore et al., 2009). Individuals may differ in their beliefs on the propriety of negative reciprocity or their abilities to reciprocate in other ways (Mitchell and Ambrose, 2007). According to the findings, certain targets’ performance may be less affected by uncivil behaviors due to their individual characteristics (Milam et al., 2009). Career resilience is an accurate description of the concept of resilience in the occupational domain (Mishra and McDonald, 2017), seeing as an individual’s ability to effectively adapt to changeable and complex occupational environments, helps individuals recover from negative experiences by changing their interpretation of adverse events (Shin et al., 2012). That is, the higher an individual’s career resilience is, the more he or she can overcome pressures and setbacks, while those with low career resilience are prone to fall into frustration.

When encountering “tit-for-tat” exchanges of social resources for incivility (Andersson and Pearson, 1999), individuals with high career resilience are capable of adjusting their mindset in a short time and are more motivated to improve their current situation (De La Rosa et al., 2016), which makes uncivil behavior less damaging to their interpersonal trust. On the other hand, individuals with low career resilience, who are not expertized in stimulating their psychological potential in the workplace, manifested maladaptation to cope with the negative impact of incivility due to the difficulties of adjusting their mindset immediately to adapt to the environment (Luthans et al., 2008). Based on the above analysis, we propose that how workplace incivility affects interpersonal trust among nurses varies with the levels of individual career resilience. In other words, we propose:

**Hypothesis 3.** Career resilience negatively moderates the relationship between workplace incivility and interpersonal trust, that is, the positive relationship is stronger when career resilience is lower rather than when it is higher.

Furthermore, this study also predicts a moderated mediation effect, with career resilience expected to serve as a buffer of the indirect effect of workplace incivility on work alienation through interpersonal trust. Individuals with low career resilience have relatively poor adaptability and psychological resilience at work, so experiencing incivility has a greater negative impact on their interpersonal trust, resulting in work alienation. In other words, the negative effect of workplace incivility through interpersonal trust on nurses’ work alienation may be mitigated when their career resilience is high. As such, we propose the following hypothesis.

**Hypothesis 4.** The indirect relationship between workplace incivility and work alienation through interpersonal trust is moderated by career resilience, such that this indirect relationship is weaker at higher levels of career resilience.

**FIGURE 1:**
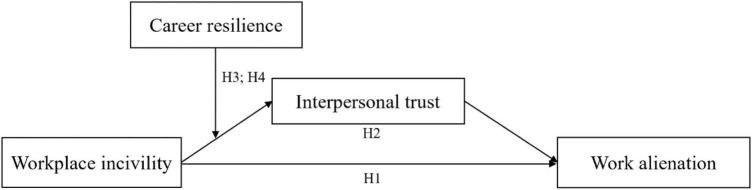
Research model.

Figure 1 shows the theoretical hypothesized model of this study.

## Materials and methods

### Sample and procedure

Our data were collected from three hospitals in Zhejiang Province, China. To minimize interruptions to hospital operations, the hospital administrators aided us in randomly selecting nurses throughout the hospital. Registered nurses employed at the hospital were encouraged to participate survey, while student nurses or nurses on orientation were excluded. All of the respondents confirmed that they had experienced incivility. Before obtaining informed consent, nurses who were eligible for this study were informed of the study purpose and procedure. Upon voluntary participation, whereas all replies were strictly private for research purposes only.

The two-wave research design was employed to reduce concerns of common method bias (Podsakoff et al., 2012). We collaborated with the hospital administration to collect data in a way that protected confidentiality and anonymity, matching participants using codes in two phases to guarantee that their identities were not revealed to the research team and that the survey data were not accessible to the hospital management. At time 1, demographics, workplace incivility, and career resilience were assessed. At time 2, survey data concerning interpersonal trust and work alienation was sent out. In total, 405 questionnaires were distributed at time 1 (May 2020) that yield 351 valid responses. After approximately 1 month (time 2, June 2020), samples who had completed the questionnaires at time 1 were asked to complete follow-up questionnaires from which 315 valid responses were eventually obtained.

Of the 315 final respondents, 31.1% were under age 26; 50.5% were between 26 and 35 years old; 16.2% were between 36 and 45 years old; and 2.2% were above age 45. Among them, 63.8% have worked for 5 years or less, 26.0% have worked for 6–10 years, and 10.2% have worked for more than 10 years. Moreover, 70.8% had obtained a bachelor’s degree or above.

### Measures

All English scales were translated and back-translated, following Brislin (1980). Three bilingual researchers translated scales originally in English. The survey was initially translated into Chinese by one bilingual, and then a second researcher double-checked the Chinese translation and resolved minor disagreements with the first translator. A third researcher then back-translated the resulting Chinese survey. The three researchers discussed and made modifications to reconcile discrepancies. The scores in each measure could be attained by calculating the mean.

#### Workplace incivility

Workplace incivility was measured by a seven-item scale developed by Cortina et al. (2001), which asked participants to indicate the frequency with which they encountered uncivil behavior from supervisors or coworkers. The scale has good reliability and validity in existing studies and is suitable for research in nursing (Andel et al., 2022). A sample item was “Paid little attention to your statement or showed little interest in your opinion.” Responses were measured with a five-point Likert scale, ranging from 1 (never) to 5 (always). The scale’s reliability was shown with a Cronbach’s alpha value of 0.93.

#### Interpersonal trust

The interpersonal trust scale was conducted using the Eleven Item scale Test developed by McAllister (1995). A sample item including questions such as “We have a sharing relationship. We can both freely share our ideas, feelings, and hopes.” The score for each item ranged from 1 (strongly disagree) to 5 (strongly agree). This scale had a Cronbach’s coefficient of 0.93.

#### Work alienation

Perceived work alienation was measured with a ten-item scale developed by Hirschfeld and Feild (2000). Nurses were asked to rate statements such as “I find it difficult to imagine enthusiasm concerning work.” The options ranged from 1 (strongly disagree) to 5 (strongly agree). The Cronbach’s alpha coefficient of the questionnaire was 0.84.

#### Career resilience

Career resilience was measured with a fourteen-item scale developed by Grzeda and Prince (1997). The rating of each item was on a five-point Likert-type scale, from 1 (strongly disagree) to 5 (strongly agree). An example item was, “Did you welcome job and organizational changes?” The Cronbach’s alpha coefficient was 0.96.

#### Control variables

In line with Bernerth and Aguinis’s (2016) guidelines, we added control variables, including age, work tenure, education, and position that altered perceptions of work alienation, in correspondence with previous research (Zhou and Long, 2011). Age was coded: 1 = 25 or below, 2 = 26–35, 3 = 36–45, 4 = 46 or above. Tenure was measured in years using three categories: 1 = 5 or below, 2 = 6–10, 3 = 10 or above. Education was coded: 1 = high school or under, 2 = vocational school, 3 = university, 4 = graduate school or above. Position was coded: 1 = registered nurse, 2 = senior nurse, 3 = supervisor nurse.

### Data analysis

SPSS 24.0 and AMOS 24.0 were used to analyze the collected data. Before proceeding on to test the main hypotheses of the study, we tested the reliability and validity of the data. Specifically, a Confirmatory Factor Analysis (CFA) and homogeneity reliability analysis were conducted to examine common method bias. In the CFA process, we constructed structural equation models to compare the fitting data of the model. Descriptive statistics were computed to describe the demographic characteristics of the participants. Pearson’s correlation analysis was used to explore the correlations among variables. A hierarchical regression analysis with a bias-corrected bootstrap technique was conducted to investigate the mediating effect of interpersonal trust in the relationship between workplace incivility and work alienation. A similar technique was applied to examine the moderating effect of career resilience in the relationship between workplace incivility and interpersonal trust. For testing moderated mediation, Hayes’s Process macros examine if the value of the moderator influences the extent of the mediation effect (Hayes, 2017). This procedure generated 95% bias-corrected confidence intervals (CIs) of these effects. The resampling value of the data was set to 5,000 resamples. The effects are considered significant at the α value of 0.05 when CIs do not include zero.

## Results

### Confirmatory factor analysis

Following the suggestion of Podsakoff et al. (2003), CFA was used to compare the fit indices of the factor models and test the discriminant validity of the model. We compared our hypothesized model (i.e., model 4, the baseline four-factor model) with a three-factor models (i.e., model 3, combining workplace incivility and interpersonal trust), a two-factor model (i.e., model 2, combining workplace incivility, interpersonal trust and career resilience), and a one-factor model combining all items (i.e., model 1, see Table 1). The CFA results suggested that our hypothesized four-factor model shows a better fit with the data (χ^2^/df = 1.56, RMSEA = 0.04, CFI = 0.95, ILI = 0.95) and better than other alternative models. The results supported the distinctiveness of the studied constructs.

**TABLE 1:** Results of confirmatory factor analysis of the measurement models.

Measurement models	χ^2^	df	χ^2^/df	RMSEA	IFI	CFI
Model 4: Four-factor	1270.79	813	1.56	0.04	0.95	0.95
Model 3: Three-factor (combined Workplace Incivility and Interpersonal Trust into one factor)	2657.35	816	3.26	0.09	0.79	0.78
Model 2: Two-factor (combined Workplace Incivility, Interpersonal Trust and Career resilience into one factor)	4384.21	818	5.36	0.12	0.59	0.59
Model 1: One-factor (combined all items into one factor)	5106.53	819	6.26	0.13	0.51	0.51

### Descriptive and correlation analysis

Table 2 contained the means, standard deviations, reliabilities, and coefficient of the study variables. The Cronbach’s alpha composite reliability (CR), average variance extracted (AVE), kurtosis, and skewness for adopted scales were meeting satisfactory levels. Correlation analysis showed that interpersonal sensitivity was significantly negatively correlated with interpersonal trust (*r* = –0.33, *p* < 0.01) and was positively correlated with work alienation (*r* = 0.30, *p* < 0.01). Interpersonal trust was significantly positively correlated with work alienation (*r* = –32, *p* < 0.01). Thus, these results preliminarily supported the subsequent regression analysis.

**TABLE 2:** Descriptive statistics and correlation analysis (*N* = 315).

	*M*	SD	1	2	3	4	5	6	7	8
Time 1										
1. Age	1.90	0.74								
2. Tenure	1.46	0.67	0.66**							
3. Education	2.67	0.75	–0.22**	–0.38**						
4. Position	1.87	0.68	0.47**	0.31**	0.19**					
5. Workplace incivility	2.63	0.88	0.09	–0.04	–0.07	–0.08	(0.93)			
6. Career resilience	3.26	0.81	–0.05	–0.12*	0.03	–0.02	0.16**	(0.93)		
Time 2										
7. Interpersonal trust	3.30	0.80	0.08	–0.01	–0.02	0.00	–0.33**	0.43**	(0.84)	
8. Work alienation	2.69	0.56	0.09	0.04	0.06	0.03	0.30**	–0.18**	–0.32**	(0.96)

*p < 0.05; **p < 0.01. M, mean; SD, standard deviation.

### Hypotheses testing

**FIGURE 2:**
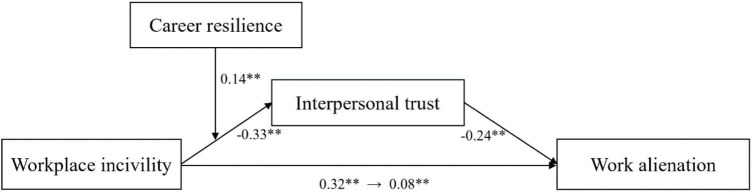
The moderated mediation model. *p < 0.05; **p < 0.01.

Table 3 and Figure 2 presented the analytical results of our hypothesized model. The control variables, age, work tenure, education, and position, were included in all the analyses.

**TABLE 3:** Regression summary.

	Interpersonal trust				Work alienation		
	M1	M2	M3	M4	M5	M6	M7
Control variables							
Age	0.03	0.07	0.06	0.04	–0.04	–0.07	–0.06
Tenure	–0.05	–0.09	–0.01	–0.02	0.10	0.13	0.11
Education	–0.03	–0.05	–0.04	–0.05	0.09	0.11	0.10
Position	0.00	–0.02	–0.04	–0.01	0.00	0.02	0.02
Independent variable							
Workplace incivility		–0.33**	–0.41**	–0.41**		0.32**	0.23**
Mediator							
Interpersonal trust							–0.24**
Moderator							
Career resilience			0.49**	0.53**			
Interaction term							
Workplace incivility × career resilience				0.14**			
*F*	0.11	7.74**	27.13**	24.95**	0.64	7.31**	9.62**
*R* ^2^	0.00	0.11	0.35	0.36	0.01	0.11	0.16
Δ*R*^2^	0.01	0.10	0.23	0.02	0.01	0.10	0.05

*p < 0.05; **p < 0.01.

Hypothesis 1 predicted a positive and direct effect of workplace incivility on nurse work alienation. Model 6 in Table 3 showed that workplace incivility was significantly related to work alienation (M6, β = 0.32, *p* < 0.01), thus supporting Hypothesis 1.

Hypothesis 2 proposed that interpersonal trust mediated the relationship between workplace incivility and work alienation. We then performed a biased-corrected percentile bootstrap method based on the above regression estimates by using the PROCESS macro (Hayes, 2017; Model 4). Results of the bootstrapping test [point estimate = 0.08, SE = 0.01, 95% CI = (0.03, 0.09)] supported that CI did not contain zero, indicating that the indirect effect of workplace incivility on work alienation through the interpersonal trust was statistically significant. Subsequently, Hypothesis 2 was supported.

We adopted hierarchical moderated regression analyses to assess the moderating effect of career resilience on the relationship between workplace incivility and interpersonal trust (i.e., Hypothesis 3). Consistent with our hypothesis, results shown from Model 4 in Table 3 suggested that the interaction between workplace incivility and career resilience was positively related to interpersonal trust (β = 0.14, *p* < 0.01), indicating that Stage 1 of the moderation of workplace incivility × career resilience was significant.

**FIGURE 3:**
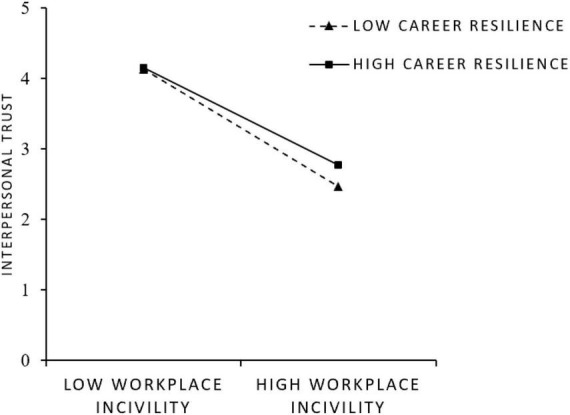
Moderating effect of interpersonal trust.

In addition, to show the moderating effect of career resilience more intuitively, we plotted the interaction effects at different levels (i.e., M + 1 SD or M–1 SD) of career resilience using the recommendation of Aiken and West (1991). Figure 3 showed that workplace incivility is more negatively related to interpersonal trust when career resilience is low rather than high, indicating that high career resilience significantly weakened the relationship between workplace incivility and interpersonal trust. Therefore, Hypothesis 3 is supported.

To examine this moderated mediation relationship proposed in Hypothesis 4, we applied Preacher et al.’s (2007) procedure. According to model 7 in PROCESS macro, the non-parametric percentile bootstrap method was used to conduct parameter estimation. The results were presented in Table 4. The indirect effect of workplace incivility on work alienation through interpersonal trust was stronger and significant at a low level of career resilience [effect size = 0.09, 95% CI = (0.04, 0.13)], but was weaker at a high level of career resilience [effect size = 0.05, 95% CI = (0.02, 0.08)]. Thus, we had further evidence to support Hypothesis 4. In addition, we used model 59 to test the moderating effect on the other two pathways (interpersonal trust on work alienation and workplace incivility on work alienation). The results showed that the 95% confidence interval contained 0, indicating that the moderation was not significant.

**TABLE 4:** Results for conditional indirect effect across levels of relational self-construction.

Condition (level of moderator – career resilience)	Indirect effect	Boot SE	Boot 95% CI
*M −* SD (Low career resilience)	0.09	0.04	(0.04, 0.13)
*M* (moderate career resilience)	0.06	0.02	(0.03, 0.09)
*M* + SD (High career resilience)	0.05	0.02	(0.02, 0.08)

## Discussions

Based on the social exchange theory, our research investigates the influencing mechanism of workplace incivility on nurses’ work alienation from the perspective of targets in the context of COVID-19.

First, this study demonstrates that workplace incivility is positively related to nurses’ work alienation. As a typically overloaded and high-risk profession, being a nurse requires an incredibly harmonious atmosphere compared with other professions with a common working environment and nature of duties. Incivility is a pervasive issue within the nursing culture (Layne et al., 2019). The occurrence of the COVID-19 pandemic has exacerbated the situation (Ghaziri et al., 2022), creating new challenges for the nurses, including frequent guideline changes on best practices for caring for patients (Markey et al., 2021) and so on. Related issues such as increased job demands and job insecurity could dramatically increase workplace incivility (Torkelson et al., 2016). Exposure to such uncivil behaviors, some nurses can be chronically troubled, shouldering a great deal of pressure (Kanitha and Naik, 2021). The accumulation of hindrance stress passes implicit messages such as a lack of work fulfillment, which reduces the engagement and passion for their work, leading to a series of conditions involving mental isolation and helplessness. This is confirmed in our study, which means uncivil encounters predict nurses’ work alienation.

Second, we revealed interpersonal trust as a critical link that partially mediates workplace incivility and work alienation. Organizational culture is crucial to the establishment of interpersonal trust (Du and Zhu, 2018). However, organizations with frequent uncivil behaviors usually fail to maintain healthy organizational culture and moral norms, which is not conducive to developing interpersonal trust. According to SET, individuals follow norms of reciprocity to seek and maintain a balance in social relations (Cropanzano and Mitchell, 2005). Balancing relations and positive connections within the dynamic social exchange process are less likely to emerge when an organization perpetrator, generally a supervisor or colleague, treats the targeted individual in a negative manner (Cropanzano et al., 2017). As workplace incivility breaks the balance of social exchange among individuals, it alienates nurses and lowers their levels of interpersonal trust. Nurses with low trust in other members tend to limit social contact in order to avoid becoming the targets of incivility, resulting in an indifferent interpersonal atmosphere of alienation in the workplace (Oyeleye et al., 2013).

Third, this study examined the moderating effect of career resilience on workplace incivility and interpersonal trust, such that workplace incivility has more substantial negative impact on the interpersonal trust of nurses with low career resilience. Meanwhile, career resilience also moderates the indirect relationship between workplace incivility and work alienation *via* interpersonal trust. Our findings on the moderating function of career resilience implied that career ability does not merely moderate the effects of perceived incivility and can influence individuals’ attitudes regarding such mistreatment. Career resilience involves not only one’s self-efficacy beliefs in coping with workplace hardship but also one’s future expectations and inclinations to overcome adversity (Sulimani-Aidan, 2017). Compared with nurses with low career resilience, those with high career resilience possess richer psychological resources, better social skills, and a more remarkable ability to adapt to the environment (Jiang et al., 2021), making it more effective for them to cope with uncivil encounters and restore psychological balance. This maintains the interpersonal trust of nurses, thereby reducing the growth of nurses’ work alienation. In this regard, the importance of tailoring remedies for the prevailing situation and the individuals involved was emphasized.

### Theoretical contributions

Firstly, this study analyzed the impact of workplace incivility on nurses’ work alienation in the context of COVID-19. Workplace incivility, deemed to create an atmosphere of staff discontent (Kavakli and Yildirim, 2022), is one of the most significant elements that lead nurses to harbor negative attitudes toward their profession (Spence Laschinger et al., 2009). This could develop into a psychological state of separation between the individual and the work. Accordingly, Arslan Yürümezoğlu and Kocaman (2019) called for investigating the impact of uncivil behavior on nurses and the interventions one could take. Our research on how incivility affects work alienation has broadened the scope of the workplace incivility literature to a certain extent. Furthermore, the findings were consistent with Zhou and Long’s (2011) view that characteristics of some professions could cause work alienation. Despite the prevalence of job alienation in nursing (Ertekin and Ozmen, 2017) and its negative consequences for nurses and organizations (Tummers and Den Dulk, 2013), our knowledge about its predictors was limited. Our study echoed the appeal for further exploration of predictors of work alienation (Amarat et al., 2019; Conway et al., 2020) and added to the scarce stream of research on work alienation in the nursing field.

Secondly, based on SET, this study verified the relationship between workplace incivility and work alienation, exploring the mediating role of interpersonal trust between them. By identifying interpersonal trust as one of the potential mechanisms underlying the negative association between experiencing incivility and job alienation, our research responded to Yao et al.’s (2021) proposal to further investigate the mediating mechanisms behind the effects of uncivil behaviors. Interpersonal trust emphasizes the mutual sharing of ideas, work-related obstacles, and a sense of mutual concern and investment (Kong et al., 2014). This sense of reciprocity is echoed in Blau’s discussion of the dynamics of social exchange (Colquitt et al., 2012). Our research further elucidated the internal mechanism of workplace incivility and revealed that social exchange processes are vital for understanding incivility’s adverse effects on work alienation. Also, our attention to interpersonal trust demonstrated that trust could be broken by transgressions (Kim et al., 2013) and responded to the appeal to explain and develop the consequences of interpersonal trust in organizational settings (Schoorman et al., 2007).

Finally, this study proved the boundary effect of career resilience, extending the research on resilience under specific domains. The results showed that career resilience could buffer the negative effects of uncivil workplace behavior on individuals, which is in response to the researchers’ call for more research into the boundary conditions of workplace incivility (Cortina and Magley, 2009). Our findings claimed that the career resilience of targets serves as boundary constraints for controlling the adverse effects of incivility and illustrated that the social exchange processes of incivility on victims varied by individual differences, consistent with Yan and Cao (2019) proposal that civility interactions focus on the degree of individual perception. Moreover, most current literature on career resilience has concentrated on testing its main effects (Wei and Taormina, 2014; Lyons et al., 2015). Our study answered the appeal to assess its moderating effects (Mishra and McDonald, 2017), in line with Meng et al. (2019), who indicated that individual resilience could improve the quality of social exchange in the workplace, providing a more nuanced understanding of career resilience.

### Implications for nursing management

The findings of this study have some implications for current nursing management. First, it calls for serious attention by medical institutions (Smith et al., 2018) and takes measures to identify, prevent and intervene in incivility, cultivate a civilized organizational atmosphere and establish relevant and effective long-term mechanisms. Secondly, efforts should be made to create a harmonious working environment. As for interpersonal conflicts that nurses cannot solve, relevant departments should take effective measures to prevent the situation from becoming more serious. Moreover, it is necessary to consider healthy interpersonal communication among medical staff to enhance their relationships. Finally, formal training programs about career resilience should be implemented, which can strengthen nurses’ perceived social support and recognition (Dyess et al., 2015). Moreover, psychological counseling and decompression training should be carried out for nurses with low career resilience, and the career resilience of nurses should be improved to deal with workplace incivility.

### Limitations and future research directions

Inevitably, this study has several limitations that point to promising future research avenues. The first limitation is that the time-lagged design limits the ability to infer causality or determine construct changes over time. Future studies could obtain longitudinal data highlighting changes and effects over a longer period. Second, the data was collected through only self-report measurements, which threatened to internal validity and caused common method bias. Future studies could integrate multiple assessment methods (i.e., experiments) with objective and qualitative data from interviews or focus groups. Third, due to restrictions caused by the COVID-19 pandemic, sample sources and quantities are constrained to minimize disruption to hospital operations. The generalizability of the conclusions was limited. In the future study, the sample size could be expanded, and additional participants with diversified backgrounds could be added. Fourth, non-independence may occur when respondents from the same hospital are clustered relative to respondents from different hospitals. More controls for non-independence could be included in future studies. Fifth, this study assessed workplace incivility without differentiating the potentially diverse impact of sources. It is, for instance, likely that supervisory uncivil behavior is more harmful than coworker incivility (Schilpzand et al., 2016). Future research could distinguish the sources and examine the distinct consequences of incivility for the affected nurses from multiple sources. Finally, since this study only considered the moderator of career resilience, the moderating effects of other organizational variables such as organizational ethical climate can also be considered in future studies.

## Data availability statement

The raw data supporting the conclusions of this article will be made available by the authors, without undue reservation.

## Ethics statement

The studies involving human participants were reviewed and approved by the Ethics Committee of Hangzhou Zhongxing Hospital. Written informed consent for participation was not required for this study in accordance with the national legislation and the institutional requirements.

## Author contributions

BX contributed in the design, write-up, and data analysis. XW designed the research. QL supervised the whole study. YH aided in the write-up and data collection. WW helped in the data collection. All authors contributed to the article and approved the submitted version.
